# A Review of Passive Micromixers with a Comparative Analysis

**DOI:** 10.3390/mi11050455

**Published:** 2020-04-27

**Authors:** Wasim Raza, Shakhawat Hossain, Kwang-Yong Kim

**Affiliations:** Department of Mechanical Engineering, Inha University, Incheon 22212, Korea; wasimkr@live.in (W.R.); msfk3742@yahoo.com (S.H.)

**Keywords:** passive micromixers, comparative analysis, Navier-Stokes equations, mixing index, pressure drop, mixing cost

## Abstract

A wide range of existing passive micromixers are reviewed, and quantitative analyses of ten typical passive micromixers were performed to compare their mixing indices, pressure drops, and mixing costs under the same axial length and flow conditions across a wide Reynolds number range of 0.01–120. The tested micromixers were selected from five types of micromixer designs. The analyses of flow and mixing were performed using continuity, Navier-Stokes and convection-diffusion equations. The results of the comparative analysis were presented for three different Reynolds number ranges: low-*Re* (*Re* ≤ 1), intermediate-*Re* (1 < *Re* ≤ 40), and high-*Re* (*Re* > 40) ranges, where the mixing mechanisms are different. The results show a two-dimensional micromixer of Tesla structure is recommended in the intermediate- and high-*Re* ranges, while two three-dimensional micromixers with two layers are recommended in the low-*Re* range due to their excellent mixing performance.

## 1. Introduction

Microfluidics is becoming more important in many chemical and biological applications [1,2,3]. Diffusion instead of turbulence governs the mixing of fluid species at the micrometer scale, and the mixing process is prolonged. Consequently an enhanced fluid mixing capability is essential for the design of micromixers. Micromixers are necessary parts of lab-on-a-chip (LOC) devices and micro-total analysis systems (μ-TAS) [4,5,6,7,8,9,10]. In various microfluidic applications, the mixing capability of micromixers may affect the overall performance of the entire systems. For example, fast mixing of cells, reagents, and organic solutions are essential in many bioengineering and biochemical systems [11,12,13]. A notable example of the mixing of reagents or organic solutions is nanomaterial synthesis [14,15]. Efficient mixing significantly improves the detection sensitivity and reduces the analysis time [16,17].

Based on the mixing mechanisms, micromixers generally come under two categories: active and passive types. In active micromixers, flow perturbation is created using external energy sources, such as magnetic fields, electric fields, and ultrasonic vibration. Additionally, active micromixers require a control mechanism, which makes the entire system more complex and creates difficulties in fabrication and operation. However, acoustofluidic devices [18,19,20] showed promising prospects for lab-on-a-chip applications and in the bio-medical diagnostic field. Sharp-edge-based acoustic active micromixers [20,21] with serpentine-like channels were recently fabricated in a simple manner and used for versatile applications. Furthermore, an active acoustic-based micromixer [18] also achieved combined pumping and mixing in a single device; in other words, it required no external source of fluid pumping.

Contrarily, no external energy source except for pumping of fluids is required in passive micromixers, and the mixing is performed by molecular diffusion and chaotic advection. Chaotic advection is generally created by modifying the microchannel geometry to reduce the diffusion length and maximize the interfacial area between the fluids by manipulating or reorganizing the fluid flow. Thus, compared with active micromixers, passive micromixers are economical, convenient, and can easily be incorporated into LOC systems [10,22,23,24]. Passive micromixers are also used as ‘concentration gradient generators’ for biological and pharmaceutical applications [25,26]. Recently, various passive mixers have been proposed to accomplish effective mixing. Chaotic flows induced by repeated perturbations of the fluid flow can significantly increase the performance of micromixers [27,28,29], and are generated by several different microchannel geometries: two-dimensional (2D) structures [30,31,32,33,34,35,36,37,38,39,40,41], three-dimensional (3D) serpentine structures [42,43,44,45,46,47,48,49], patterned groove structures [29,50,51,52,53,54,55,56,57,58], 2D and 3D split-and-recombination (SAR) structures [59,60,61,62,63,64,65,66,67,68,69,70,71,72,73,74,75,76,77,78,79,80,81], and two-layer crossing channels [82,83,84,85,86,87,88,89].

Several review articles have introduced a variety of passive micromixers with their mixing mechanisms [6,24,90,91,92,93,94,95]; these micromixers have various dimensions and working conditions (e.g., Reynolds number). However, quantitative comparisons of their mixing performance are rarely found in the literature, even though designers would benefit from the information on mixing performance of different micromixers under the same geometric and working conditions. Such information will assist in the selection of suitable designs to meet specific requirements for different processes [92]. 

Quantitative comparisons have been performed only for specific types of passive micromixers. Falk and Commenge performed a comprehensive study of conventional T-type micromixers and micromixers based on the concept of SAR or multi-lamination through the Villermaux-Dushman test reaction [96]. They analyzed and compared the mixing efficiencies of the micromixers while considering Reynolds number and power dissipation per unit mass of the liquid. Viktorov et al. presented a comparative analysis of three passive micromixers (tear-drop, Y-Y, and H-C micromixers) in a wide range of Reynolds numbers [97]. They conducted a numerical simulation and experimental analysis to evaluate the mixing performance at Reynolds numbers ranging from 1 to 100. Bošković et al. analyzed and compared the characteristics of the residence time in three different passive micromixers (micromixers with 3D serpentine structure, staggered herringbone grooves, and split and recombine (SAR) structure) [98]. The microstructures with similar channel designs were analyzed across a wide range of Reynolds numbers (0.3 ≤ *Re* ≤ 110). 

As mentioned above, a variety of micromixer designs have been developed so far, but no one has reported a quantitative evaluation of the micromixers operating at the same conditions, which is necessary for the selection of effective micromixers under different flow conditions in various microfluidic applications. Therefore, in the present work, a review of a wide range of existing passive micromixers is presented and a comparative analysis of selected micromixers was performed under the same working fluids, Reynolds number, and axial channel length for a quantitative comparison among them. For the comparative analysis, ten micromixers were selected among the high-performance micromixers found in the literature, which cover five typical micromixer designs. These micromixers achieved efficient mixing with different mixing mechanisms or their combinations, as explained in Section 2. The numerical analyses of mixing and fluid flow were performed using Navier-Stokes and advection-diffusion equations for momentum and mass transports, respectively. The comparison was performed in a Reynolds number range of 0.01–120. 

## 2. Mixing Mechanisms of Micromixer Types and Selected Micromixers 

**Figure 1:**
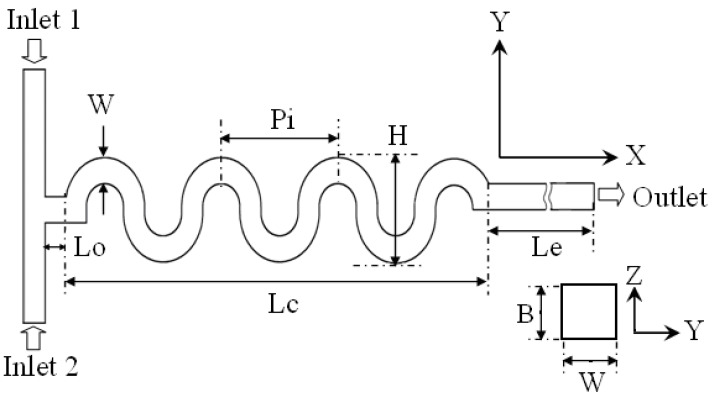
Curved micromixer: M-1 (no. of mixing units used: 8). Reproduced with permission from [40].

**Figure 2:**
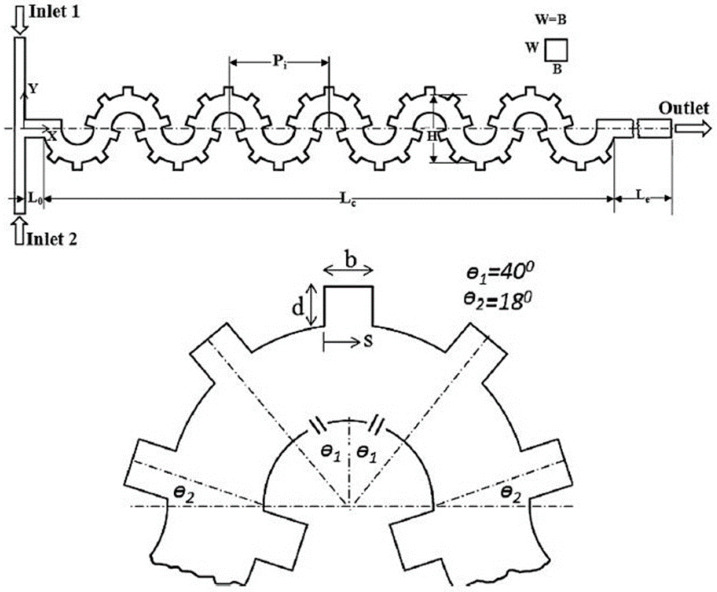
Curved micromixer with rectangular grooves: M-2 (no. of mixing units used: 8). Reproduced with permission from [41].

**Figure 3:**
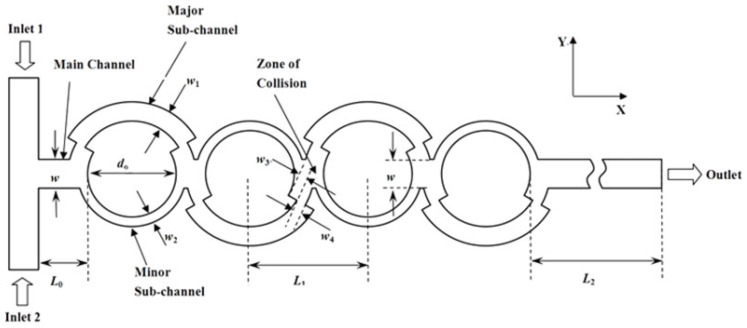
Modified P-SAR micromixer with dislocation sub-channels: M-3 (no. of mixing units used: 4). Reproduced with permission from [68].

**Figure 4:**
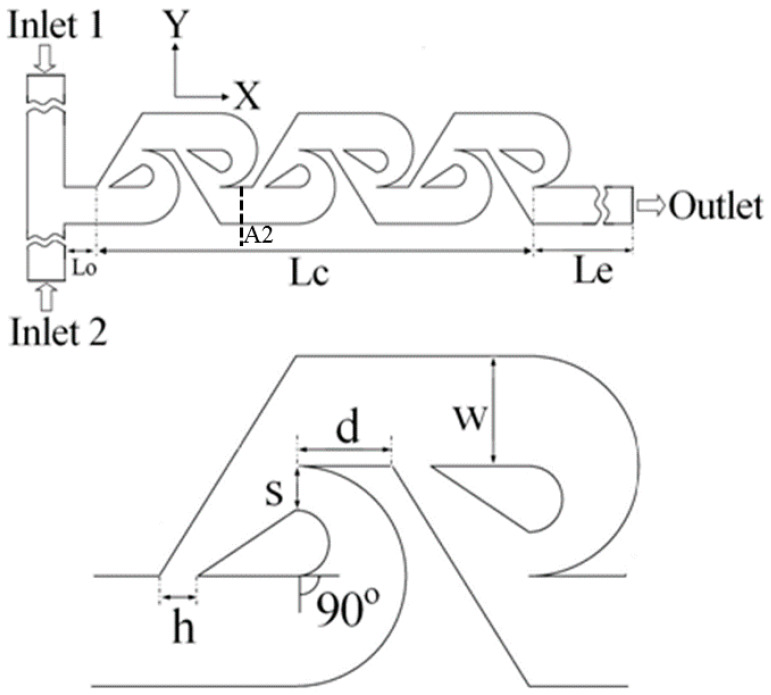
Modified Tesla micromixer: M-4 (no. of mixing units used: 6). Reproduced with permission from [77].

**Figure 5:**
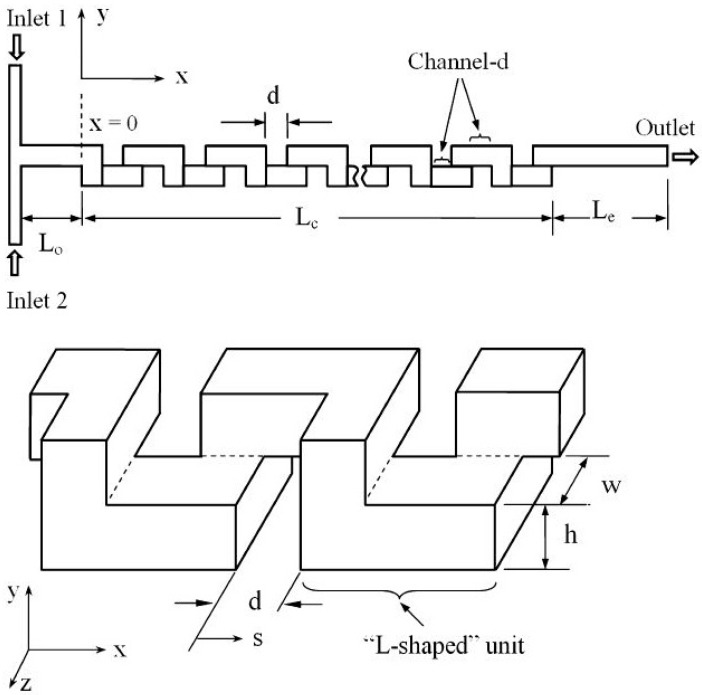
3D serpentine micromixer: M-5 (no. of mixing units used: 8). Reproduced with permission from [45].

**Figure 6:**
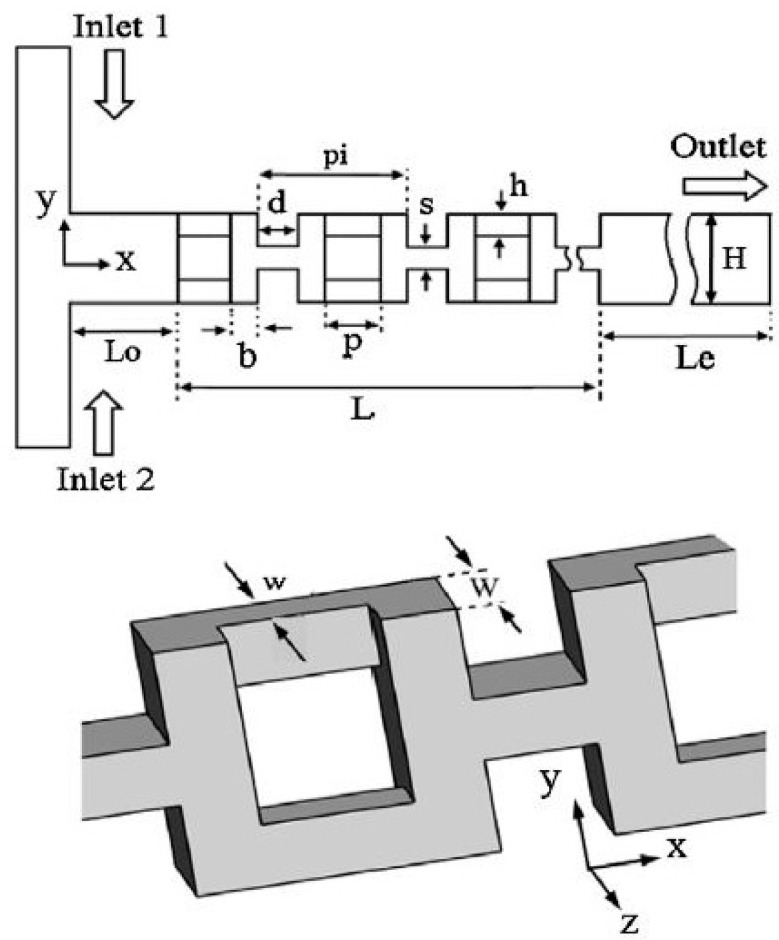
3D serpentine SAR micromixer: M-6 (no. of mixing units used: 18). Reproduced with permission from [59].

**Figure 7:**
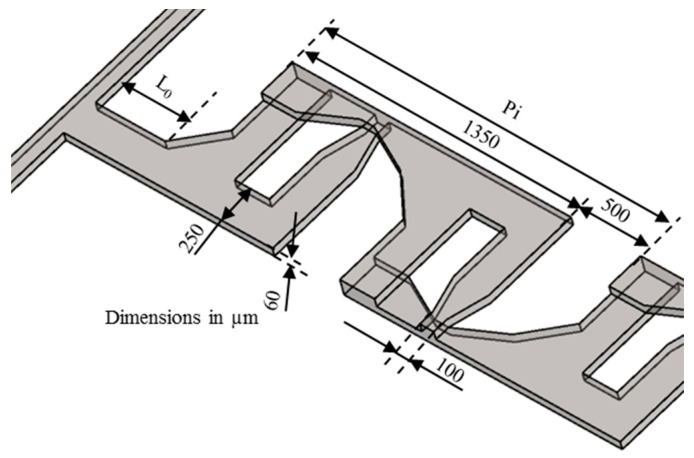
Improved serpentine lamination micromixer: M-7 (no. of mixing units used: 2) [81].

**Figure 8:**
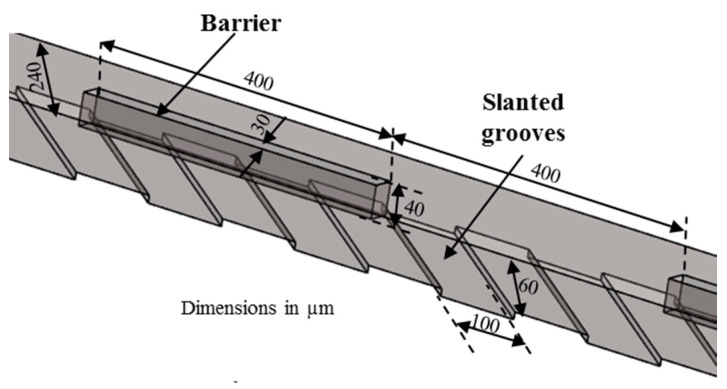
Barrier embedded chaotic micromixer: M-8 (no. of mixing units used: 6) [58].

**Figure 9:**
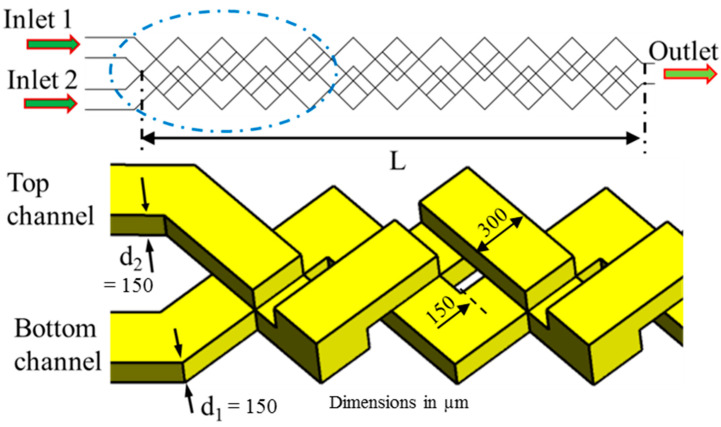
Chaotic micromixer with two-layer crossing microchannels: M-9 (no. of mixing units used: 8). Reproduced with permission from [89].

**Figure 10:**
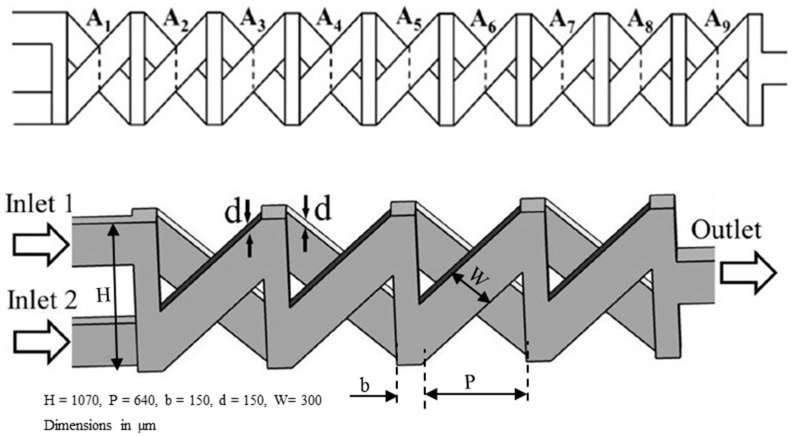
Chaotic micromixer with two-layer serpentine crossing microchannels: M-10 (no. of mixing units used: 6). Reproduced with permission from [87].

In the last two decades, a number of passive micromixers involving different microchannel designs have been proposed, as introduced in the previous section. The microchannel designs can be categorized into five types, as shown in Table 1. For the quantitative comparison in this work, ten representative micromixers (M-1 to M-10) were selected as shown in Table 2, and Figure 1, Figure 2, Figure 3, Figure 4, Figure 5, Figure 6, Figure 7, Figure 8, Figure 9 and Figure 10 show that their schematics. M-1 to M-4 are 2D planar designs, and M-5 to M-10 are 3D designs. To compare the mixing performances at an equal axial length, which refers to the length of the channel in the *x*-direction between the start of the mixing unit and the micromixer exit, the number of mixing units in each micromixer was changed from the original number as indicated in each figure caption. The dimensions of the mixing unit in each micromixer were the same as those in its original design. The mixing capability was evaluated at each micromixer exit (5050 µm (*L_t_*) downstream of the start of the mixing unit) for the comparison. 

### 2.1. 2D Designs Using Serpentine, Spiral, and Curved Helical Channels (Type 1)

2D planar micromixer designs have an advantage of simplicity in the fabrication compared to the complex 3D designs. Mixing in 2D serpentine, spiral, and curved helical channels [30,31,32,33,34,35,36,37,38,39,40,41] mainly depends on the advection caused by the secondary flow or Dean vortices created by the inertia force. The performance of this type (type 1 in Table 1) of micromixers improves as the Reynolds number increases due to the dependence of secondary flow/Dean vortices on Reynolds number [78]. Hossain et al. [40] and Alam and Kim [41] conducted numerical investigations of mixing in 2D planar micromixers, M-1 (Figure 1) and M-2 (Figure 2), respectively. Hossain et al. [40] estimated the mixing performance of three serpentine passive micromixers (square-wave, zigzag, and curved shape microchannels) across a wide range of Reynolds numbers (0.267–267). Alam and Kim [41] introduced rectangular grooves on the sidewalls of a curved serpentine channel to evaluate mixing in a Reynolds number range of 0.5–90. 

### 2.2. 2D Designs with SAR Structures (Type 2)

A variety of planar SAR micromixers (type 2 in Table 1) involving multi-lamination and inertial flow have been developed [68,69,70,71,72,73,74,75,76,77,78,79]. The SAR micromixers generate multi-laminating flow patterns successively with the three underlying flow mechanisms of splitting, recombination, and rearrangement [6]. Dean and expansion vortices are generated through curved channels and expansion-contraction at high Reynolds numbers [70,71,76]. 

Ansari et al. [76] proposed and analyzed a micromixer using asymmetrical splits and collisions of fluid streams. The lowest mixing performance was obtained with uniform sub-channel widths representing balanced collision over a range of Reynolds numbers. Induced Dean vortices at the interfaces in the curved sub-channels and SAR were found to enhance the mixing performance. Xia et al. [70] designed an asymmetric SAR micromixer with a fan-shaped cavity to achieve efficient mixing by the synergistic effect of expansion vortices and Dean vortices in the fan-shaped cavity along with unbalanced collision in the recombination zone. The selected micromixer, M-3 [68] shown in Figure 3, represents a planar asymmetric SAR (P-ASAR) design with outward protruded sub-channels, which is an improved form of a previous micromixer [76]. In this micromixer, the mixing performance was enhanced by the synergistic effect of unbalanced inertial collisions, expansion vortices, and Dean vortices on mixing. 

Xia et al. [74] designed and fabricated a 2D planner micromixer consisting of a series of gaps and baffles in a simple microchannel. A sudden contraction provided by a gap accelerates the fluid stream and produces symmetrical expansion vortices. The accelerated fluid stream is separated by a baffle, and the same flow pattern is repeated. The synergistic effects of abrupt contraction and expansion, multiple SAR, and multiple secondary vortices increase the interfacial area of the fluids, resulting in excellent mixing performance. Hong et al. [72] proposed an innovative micromixer with a 2D modified Tesla structure (M-4) shown in Figure 4, which takes advantage of the Coanda effect. The splitting and reuniting of the fluids effectively reduce the diffusion path between the fluid streams. The structure causes chaotic flow by the collision of the fluid streams on redirection, and improves mixing. Hossain et al. [77] improved the efficiency of a modified Tesla micromixer through an optimization.

### 2.3. 3D Design with Serpentine and/or SAR Structures (Type 3)

Several micromixers involving 3D serpentine and/or SAR structures [42,43,44,45,46,47,48,49,59,60,61,62,63,64,65,66,67,80,81] (type 3 in Table 1) have been proposed. The 3D serpentine path induces a stirring flow at each bend and generates a secondary flow to enhance mixing [43,45]. The SAR structure provides lamination that decreases the diffusion path and enhances mixing at low Reynolds numbers [65]. The secondary flow combines with the axial flow, and creates chaos by stretching and folding the fluid interface [47]. 

Ansari and Kim [45] investigated the effects of flow and geometric parameters on the mixing performance of an L-shaped 3D serpentine micromixer (M-5) shown in Figure 5. Hossain and Kim [59] proposed a 3D serpentine SAR micromixer composed of O- and H-shaped units (M-6) shown in Figure 6. The O- and H-structures split and recombine the fluid streams repeatedly. Continuous splitting and recombining of the fluid streams generate chaotic mixing. Kim et al. [80] proposed a chaotic-mixing-based serpentine lamination micromixer (SLM) composed of F-shaped mixing units. The SLM structure combines two general chaotic mixing mechanisms: SAR induced by the mixing segments and chaotic advection induced by the overall 3D serpentine channel path. Park et al. [81] proposed a geometrical modification of SLM to improve the mixing efficiency. Figure 7 shows the improved SLM (ISLM) (M-7), where the original F-shaped mixer is altered at the recombination region. The reduced cross-sectional area enhances the vertical lamination by enhancing the local advection, consequently improving the mixing performance. 

### 2.4. 3D Design with Patterned Grooves (Type 4) 

Patterned grooves on the channel wall were also used to promote mixing in microchannels [29,50,51,52,53,54,55,56,57,58] (type 4 in Table 1). The grooves induce 3D helical flow in the microchannels, which promotes mixing. Kim et al. [58] designed a micromixer with rectangular barriers on the top of the slanted grooves (M-8) shown in Figure 8. The periodically located barriers on the top wall are capable of creating velocity fields characterized by two elliptic points and a hyperbolic point alternately within the helical motion produced by the grooves at the bottom, which results in enhancement of chaotic mixing. 

### 2.5. 3D Designs with SAR Two-Layer Crossing Channels (Type 5)

3D micromixers with SAR two-layer crossing channels (type 5 in Table 1) have achieved remarkable mixing performance [82,83,84,85,86,87,88,89]. The crossing channels produce chaotic flow due to repeated splitting, stretching, rotating, and folding processes. A saddle-shaped flow structure is produced despite low Reynolds number. Hence, the chaotic flow inside the crossing channels does not depend on the fluid inertia [84]. 

Xia et al. [84] designed a chaotic micromixer (M-9) using 3D X-shaped crossing channels (TLCCM) shown in Figure 9. The micromixer exhibited an outstanding mixing efficiency of 96% at a low Reynolds number (*Re* = 0.2). Hossain et al. [89] further analyzed the responses of the flow structure and mixing performance to the variations in geometric parameters of TLCCM.

Recently, Hossain et al. [87] designed a chaotic micromixer with two-layer serpentine crossing microchannels (M-10) based on the mixing mechanism of TLCCM (Figure 10) to improve the mixing capability at low Reynolds numbers. This micromixer consists of two layers of serpentine channels. The bottom and top layers contain a series of N- and inverse N-shaped segments, respectively. The fluid streams are interconnected at the vertical sections and the intersections of the crossing channels. The fluid flow in successive mixing modules produces chaotic advection through continuous splitting, recombination, enlarging, and folding of the fluid streams.

## 3. Analysis Methods 

The flow inside the microchannel was assumed to be steady, incompressible, laminar, and Newtonian. The continuity (Equation (1)), momentum (Equation (2)), and convection-diffusion (Equation (3)) equations were solved numerically for the flow and mixing analysis. The CFD software ANSYS CFX 15.0^®^ [99] employs a coupled solver and finite volume technique to discretize Equations (1)–(3):

(1) $$\nabla \cdot \vec{V}=0$$

(2) $$\left(\vec{V}\cdot \nabla \right)\vec{V}=-\frac{1}{\rho }\nabla P+\nu \nabla^{2}\vec{V}$$

(3) $$\left(\vec{V}\cdot \nabla \right)C=D\nabla^{2}C$$

where $\vec{V}$, *C*, *D*, *ρ*, *P*, and *ν* are the velocity, dye concentration, diffusion coefficient, density, pressure, and kinematic viscosity, respectively.

A finite-volume-based commercial code ANSYS CFX 15.0^®^ [99] has been used to solve the governing differential equations. Tetrahedral meshes were generated using ICEM CFD^®^ for the grid systems. The boundary conditions were the uniform velocity profiles at the inlets, zero static pressure at the outlets, and no-slip conditions at the channel walls. Dye-water solution and water, both having the properties of water at 25 °C (dynamic viscosity: 8.8 × 10^−^^4^ kg/m·s; density: 997 kg/m^3^) were used as the working fluids. The numerical solution was assumed to be converged as root-mean-squared residual values for momentum and mass fraction reach a value less than 1.0 × 10^−6^. The diffusivity constant of the solution was fixed to be 1.0 × 10^−10^ m^2^/s. Reynolds number was calculated using the hydraulic diameter and average velocity at the inlets. 

The numerical methods same as used in the present work, were validated comparing with experimental results for a variety of micromixers in previous works [40,41,45,54,55,57,59,69,75,76,77,79,87,88,89].

The mixing index was estimated by calculating the variance of concentration on a particular transverse plane normal to the fluid flow. The mass fraction variance was determined as:

(4) $$\sigma =\sqrt{\frac{1}{N}\overset{N}{\underset{i=1}{\sum }}{\left(c_{i}-{\bar{c}}_{m\;}\right)}^{2}}$$

where *N* denotes the number of data points on the cross-sectional plane, ${\bar{c}}_{m\;}$ is the optimal mass fraction, and $c_{i}$ is the mass fraction at a point i. The mixing index was defined as:

(5) $$M=1-\frac{\sigma }{\sigma_{\max}}$$

where σ_max_ is the maximum variance over the data range. The mixing index ranges from zero (wholly separated fluid streams) to unity (complete mixing).

The other mixing performance parameter, mixing cost (*MC*) [100] that takes pressure drop into account along with the mixing index was defined as follows: where *N* denotes the number of data points on the cross-sectional plane, c_m_ is the optimal mass fraction, and c_i_ is the mass fraction at a point *i*. And, the mixing index was defined as:

(6) $$\mathrm{mixing}\;\cos t\equiv \frac{M}{\Delta P}$$

where *ΔP* denotes the pressure drop. A high value of *MC* indicates an efficient micromixer.

## 4. Results and Discussion 

### 4.1. Grid Refinement Test

Optimal numbers of computational grid nodes were determined after performing exhaustive grid refinement tests at *Re* = 40 for all the micromixers. Mixing index at the exit was selected as an indicator for choosing the optimal grids. The number of grid nodes from 3.86 × 10^5^ to 2.55 × 10^6^ were tested in the grid refinement tests. The optimum numbers of nodes that were selected for the ten micromixers varies from 1.55 × 10^6^ to 2.21 × 10^6^, as listed in Table 3. 

### 4.2. Quantitative Comparisons in Different Reynolds Number Ranges 

The examined range of Reynolds numbers (*Re* = 0.01–120) was divided into three sub-ranges: low-*Re* (*Re* ≤ 1), intermediate-*Re* (1 < *Re* ≤ 40), and high-*Re* (*Re* > 40) ranges. One of the primary purposes of the present comparative analysis was to find efficient micromixers in each *Re* range. Evaluated values of mixing index (*M*), pressure drop (ΔP), and mixing cost (*MC*) for each micromixer are presented in Table 4, Table 5 and Table 6, respectively.

**Figure 11:**
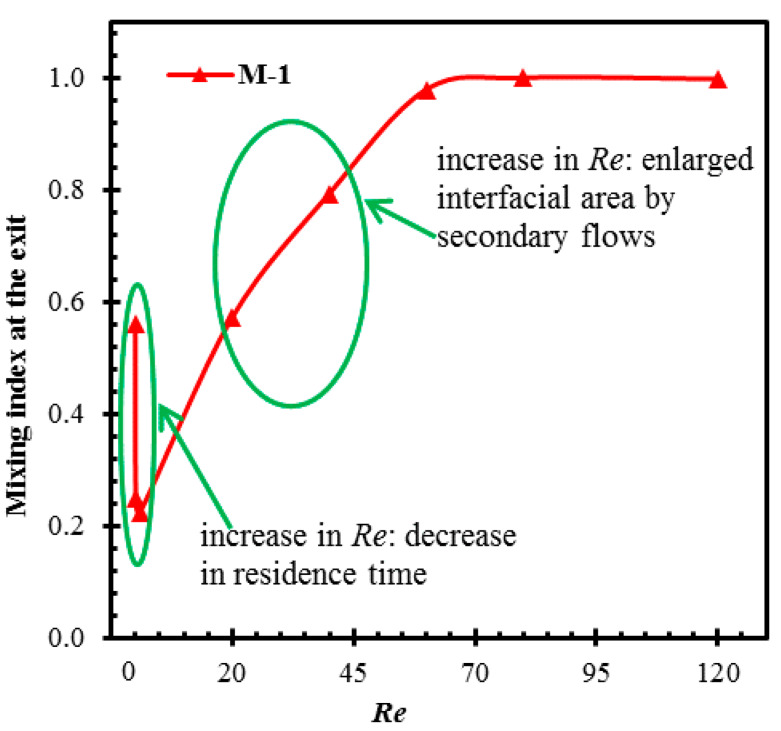
Variation of the mixing index at the exit with Reynolds number in a curved micromixer [40].

Figure 11 shows the general trend of the mixing index variation with Reynolds number for the curved micromixer, M-1 [40]. At low *Re* (*Re* ≤ 1), low velocity of the fluid stream causes a long residential time of the fluids within the microchannel, which provides sufficient time for diffusive mixing. The mixing deteriorates rapidly as Reynolds number increases due to the reduction in the residential time, reaching a minimum at around *Re* = 1, where the residential time is inadequate, and the transverse flow is still ineffective for generating the secondary flow. Thus, the mixing index remains at a low level. However, beyond this *Re*, the residence time reduces further, but the secondary flow becomes active. Thus, mixing starts to increase with Reynolds number. The specific variation of the mixing index with Reynolds number strongly depends on micromixer configuration, but the trends are similar for different passive micromixers.

#### 4.2.1. Mixing in Low-*Re* Range (Re ≤ 1) 

In the low-*Re* range, mixing in passive micromixers is limited by molecular diffusion. Therefore, the mixing in this range mainly depends on the residential time of the working fluids in the micromixer. Mechanical stirring is not an effective method for enhancing mixing [36] because the secondary flow is hardly induced in this range. Thus, it is very challenging for researchers to design an efficient micromixer at this low-*Re* range. 

It is found from Table 4 that, among the 2D micromixers (M-1 to M-4), the mixing indices at the exits of the curved micromixer (M-1) and the curved micromixer with rectangular grooves (M-2) are higher than the others at *Re* = 0.01 (M = 0.560 and 0.554, respectively). Interestingly, in this Reynolds number range, M-1 shows similar mixing performance as M-2. This is because, at low Reynolds numbers, mixing depends on molecular diffusion, and thus, geometric modification of the 2D planar micromixer is not effective in enhancing the mixing. The mixing index strongly depends on the time for which the working fluids remain in the micromixer. Hence, the mixing indices of these micromixers decrease as the Reynolds number increases up to 1. This trend does not only apply to the 2D micromixers but also to most of the tested micromixers. 

The SAR micromixers (M-3 and M-4) generally show lower mixing than the micromixers with 2D serpentine structures (M-1 and M-2) in this range. However, the pressure drops are much lower in SAR micromixers, as shown in Table 5. It is observed that M-4 shows a 16.9% lower mixing index at the exit with a 65% lower pressure drop, as compared to M-1 at *Re* = 0.01. Among the 2D mixers, M-1, M-2, and M-4, showing high mixing indices, M-4 represents the highest *MC* values in the low-*Re* range, as shown in Table 6. M-3 shows the worst mixing performance among the tested micromixers in this range. 

As shown in Table 4, the 3D micromixers using SAR with two-layer crossing channels (M-9 and M-10) achieve remarkable mixing performance at low Reynolds numbers. M-9 and M-10 show mixing indices over 0.90 in the entire low-*Re* range, while M-7 is only the micromixer which shows a mixing index over 0.90 (at *Re* = 0.01) in this *Re* range among the remaining micromixers. This is due to the generation of saddle-shaped flow structure in M-9 and M-10 at all Reynolds numbers, while in M-7, mixing decreases with the increase in Reynolds number due to diffusion dominant mixing. M-8 shows the lowest mixing indices among the 3D micromixers, which are even lower than those of some 2-D micromixers. This indicates that the mixing relying on elliptic and hyperbolic points generated through alternating barriers above the groove on the walls will require longer channel length for complete mixing.

Surprisingly, M-9 and M-10 also show the least pressure drops among the tested micromixers in the low-*Re* range, as shown in Table 5. Therefore, M-9 and M-10 show the highest MC values among the tested micromixers in the whole low-*Re* range (Table 6). In all the tested micromixers, MC decreases with increasing Reynolds number in this *Re* range. The micromixer M-7, which shows a not-much-lower mixing index than M-9 at *Re* = 0.01, shows about a 13-times-higher pressure drop with a 93% lower *MC* value at the same *Re*. Thus, the structure of 3D SAR with two-layer crossing channels is proved to be efficient in both enhancing mixing and reducing pressure drop in the low-*Re* range. 

**Figure 12:**
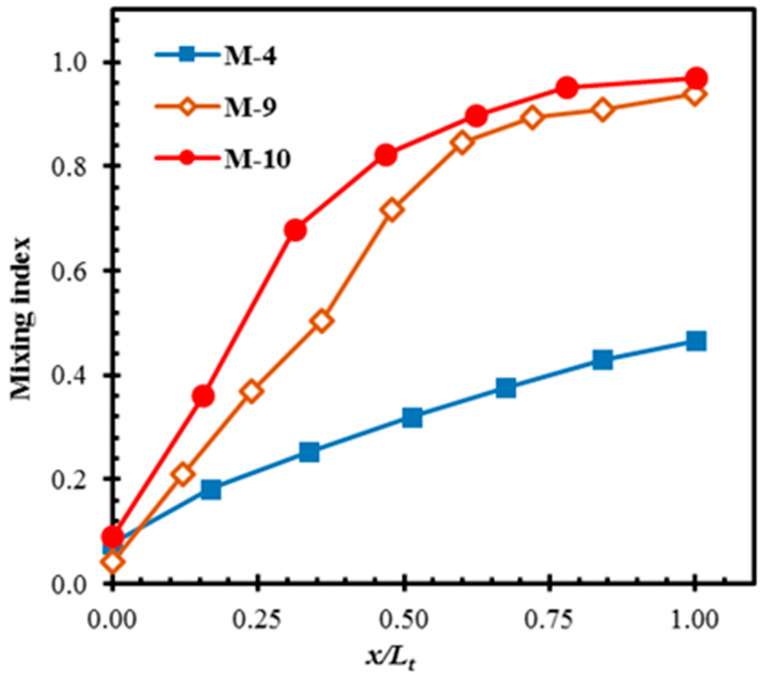
Developments of mixing along the length of the micromixers at *Re* = 0.01.

Figure 12 shows the developments of the mixing for M-4, M-9, and M-10 at *Re* = 0.01. The 2D micromixer (M-4) shows relatively slow development of mixing compared to the 3D micromixers (M-9 and M-10). Although M-9 and M-10 attain almost similar mixing at the exit, M-10 shows much faster development rate near the inlet of the micromixer. M-9 and M-10 reach a mixing index over 0.80 within 50% of the total length. M-9 attains the mixing index equal to the mixing index at the exit of M-4, within 35% of its length. This emphasizes that the 3D micromixer with an even slow development rate outperforms the 2D micromixer.

#### 4.2.2. Mixing at Intermediate Reynolds Numbers (1 < Re ≤ 40)

In the intermediate-*Re* range, the fluid inertia starts to increase with Reynolds number, and generates secondary flows that play a dominant role in mixing enhancement. Hence, an increase in Reynolds number results in an increase in mixing index in most of the cases shown in Table 4.

Among the 2D micromixers, the mixing index at the exit of the Tesla micromixer (M-4) is highest at *Re* = 20 and 40, and M-4 achieves almost perfect mixing (M = 0.999) at *Re* = 40. In this micromixer, with the increase in Reynolds number, the collision of fluid streams on rejoining is strengthened, which enhances chaos in the flow and hence the mixing [72,77]. It is also observed that the M-2 shows higher mixing than M-1 at these Reynolds numbers. This is due to the increase in secondary flow caused by the grooves in M-2 [41]. At *Re* = 20 and 40, M-2 shows 21.3% and 8.2% higher mixing indices than M-1, and M-4 shows 27.2% and 16.5% higher mixing indices than M-2, respectively. It is also observed that M-4 shows much lower pressure drops than M-1 and M-2. M-4 shows 46.8% and 26.3% lower pressure drops with 139.1% and 58.2% higher *MC* values than M-2 at *Re* = 20 and 40, respectively. Among the 2-D micromixers, M-3 shows the lowest mixing indices (Table 4) in the intermediate-*Re* range, but shows the highest MC values (Table 6) due to the lowest pressure drops (Table 5). Mixing in M-3 depends upon the unbalanced inertial collision. Hence, the lowest mixing indices in M-3 can be attributed to insufficient inertial force to cause an effective collision of the fluid streams that enhances chaos in the recombination zone. It also highlights that the flow instability in the inertia-based micromixers occurs at different Reynolds numbers depending upon the microchannel designs. Hence, these micromixers can be used in different *Re* range depending upon the efficient mixing range. 

Among the 3D micromixers, most of the micromixers perform well in intermediate-Reynolds number range except M-7 and M-8. This indicates that locally accelerated advection due to the narrowing of flow path along with SAR mechanism in M-7 and the alternating velocity field creating elliptic and hyperbolic points due to the barrier and groove configuration in M-8 are not as effective as mixing mechanisms for other micromixer designs. Especially, M-5 and M-6 show nearly perfect mixing (M = 0.999) in the whole intermediate-*Re* range. In this *Re* range, stretching and folding of the fluid interfaces is developed due to the transverse flows induced by inertial forces. It causes enlargement of the fluid interfacial area for diffusion, thereby promotes mixing [45,59]. The 3D swirling flow along with the vortical flow due to serpentine channel is present in M-7 as discussed in [81], showing 26.7% lower mixing index than M-5 and M-6 at *Re* = 40.

The structures of 3D SAR with two-layer crossing channels (M-9 and M-10) achieve over 90% mixing in the intermediate-*Re* range even though it does not show the best mixing indices among the tested cases as in the low-*Re* range. Although M-5 and M-6 show the same mixing indices, the pressure drops in M-6 are approximately 22 times higher as compared to M-5 at *Re* = 20 and 40, and thus M-5 has much higher MC values than M-6. M-10 shows 9.8% and 7.0% lower mixing indices and 84.0% and 86.0% lower pressure drops compared to M-5 at *Re* = 20 and 40, respectively. M-10 shows the highest MC values among the tested micromixers, which are 5.6 times and 6.6 times higher than those of M-5 at *Re* = 20 and 40, respectively. 

**Figure 13:**
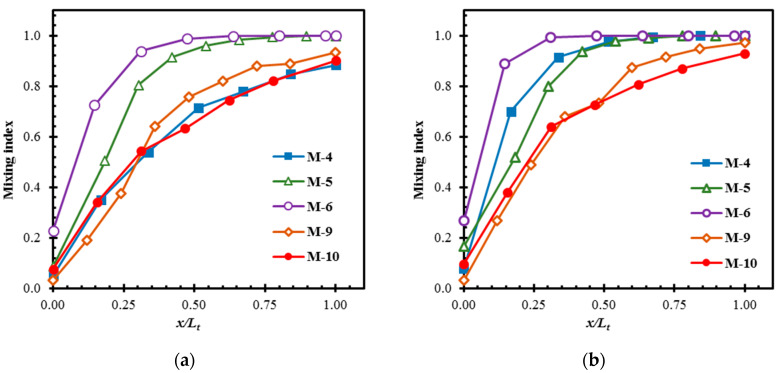
Developments of mixing along the length of the micromixers: (**a**) *Re* = 20 and (**b**) *Re* = 40.

The developments of mixing in M-4, M-5, M-6, M-9, and M-10 at *Re* = 20 and 40 are shown in Figure 13a,b, respectively. At *Re* = 20, M-4, M-9, and M-10 show similar development rates, while M-5 and M-6 show higher development rates near the micromixer inlet. At *Re* = 40, in addition to M-5 and M-6, M-4 also shows a higher rate of development as compared to M-9 and M-10. M-5 and M-6 achieve almost complete mixing at 50% and 65% of the total length, respectively, at *Re* = 20. At *Re* = 40, M-6 also shows the highest development rate and attains almost complete mixing around 30% of the length. M-4 achieves a mixing index over 0.80 in less than 25% of the length and almost complete mixing at 65% of length, whereas M-9 and M-10 achieve a mixing index over 0.8 within 60% of their lengths at *Re* = 40. M-4, M-5, and M-6 show higher increases in the development rate with the increase in Reynolds number than M-9 and M-10. The non-dependency of mixing on the inertia in M-9 and M-10 is consistent with the observation in [84].

#### 4.2.3. Mixing at High Reynolds Numbers (Re > 40)

At high Reynolds numbers, transverse flows (Dean vortices or secondary flow) become stronger, and mixing is established at a faster rate by stretching and folding of the interfacial area. Increasing the inertial force of the fluids is one of the easiest ways to enhance mixing at high-*Re* range. At the highest Reynolds number (*Re* = 120), almost all the micromixers except M-8 show excellent mixing performance. And, M-4, M-5, and M-6 attain almost complete mixing in the whole high-*Re* range. 

The quantitative comparison in Table 4 demonstrates that even simple 2D serpentine micromixers (M-1 and M-2) show more than 97% mixing at *Re* = 60, and more than 99% mixing at *Re* = 80 and 120. M-3 having asymmetrical SAR structure reaches a mixing index of 0.89 at *Re* = 120. From Table 5, it is observed that M-1 and M-2 show 7.1% and 20.6% reduction in pressure than those of M-4 at *Re* = 80 and 120, respectively. Hence, M-4 shows 7.4% and 20.5% lower *MC* values than those of M-2 at *Re* = 80 and 120, respectively, in Table 6. However, it shows 11.6% higher *MC* than that of M-2 at *Re* = 60. M-3 shows the highest MC values among the 2-D micromixers due to the lowest pressure drops.

The 3D micromixer M-7 also reaches a mixing index over 0.9 at *Re* = 80. There are minimal variations among the mixing indices of the 3D micromixers in this *Re* range if M-7 and M-8 are excepted. However, M-5 and M-6 achieve complete mixing at the expense of a high pressure drops. M5 and M-6 show approximately 7.5 times and 168 times higher pressure drops, respectively, as compared to M-10 at *Re* = 60. Hence, M-10 shows approximately 7.5 times higher *MC* as compared to M-5 in this *Re* range. The worst mixing performance among the tested micromixers is obtained by M-8. 

**Figure 14:**
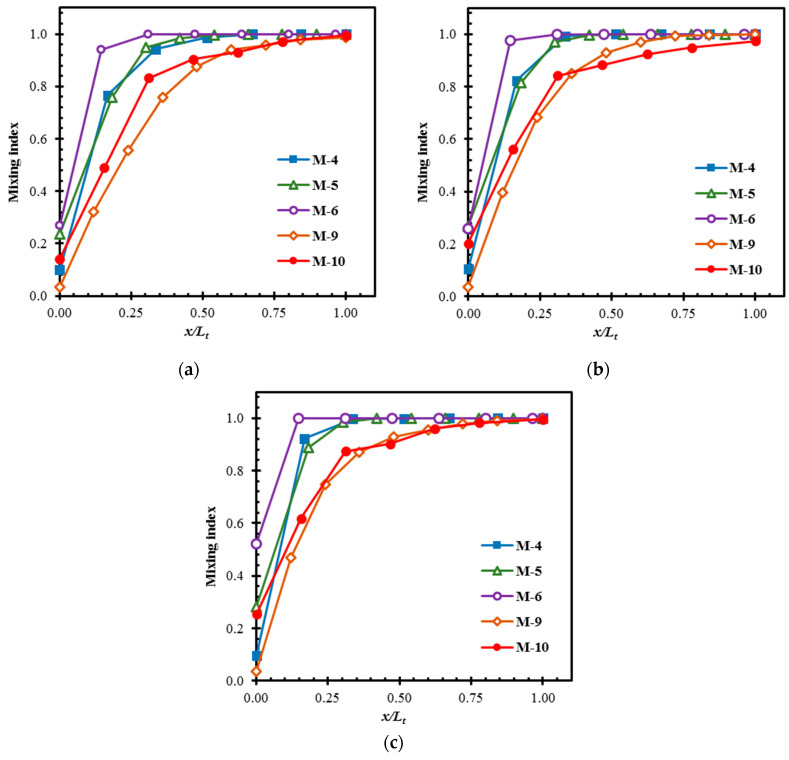
Developments of mixing along the length of the micromixers: (**a**) *Re* = 60, (**b**) *Re* = 80 and (**c**) *Re* = 120.

Figure 14 shows the developments of mixing in M-4, M-5, M-6, M-9, and M-10 at different Reynolds numbers. The rates of development of mixing near the inlets of M-4, M-5, and M-6 are found to be higher than those of M-9 and M-10 in this *Re* range. M-4 shows the highest development rate, and M-5, and M-6 show almost the same developments of mixing in the whole high-*Re* range (*Re* = 60, 80, and 120). As Reynolds number increases, the development rates of these three micromixers generally increase. M-4 achieves almost complete mixing within 15–30% of the length, and M-5 and M-6 achieve it within 30–50% of the length in this *Re* range. We found that 50% of the length of M-5 and M-6 where complete mixing is achieved, still show approximately 3 times and 84 times higher pressure drops, respectively, compared to M-10 at *Re* = 60. M-10 shows approximately 3.4 times higher *MC* as compared to 50%-length of M-5 at this Reynolds number. M-10 shows slightly better mixing rate than M-9 at *Re* = 60. M-9 and M-10 show similar trends of the mixing development especially at *Re* = Re–120, where the mixing improves continuously until the exit is approached. 

#### 4.2.4. Comparison of Velocity and Concentration Fields between M-4 and M-10

**Figure 15:**
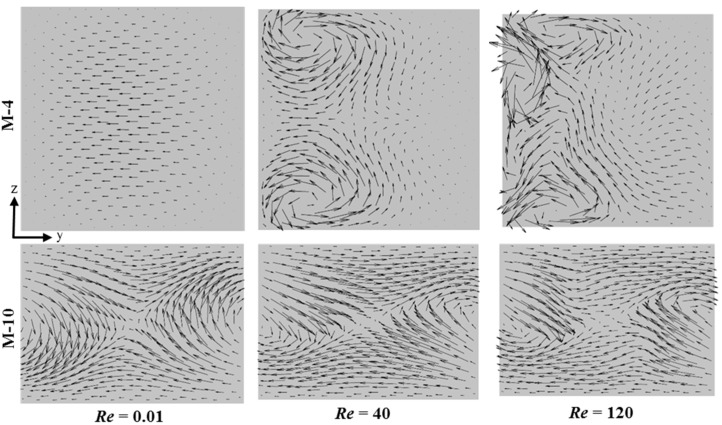
Velocity vectors in M-4 and M-10 at *x/L_t_* = 0.16 (plane A2 in Figure 4 and Figure 10) at different Reynolds numbers.

For M-4 and M-10, which show the best mixing performances among the 2D and 3D micromixers, respectively, flow structures and concentration distributions were compared at different Reynolds numbers. Figure 15 shows the flow structures on a y-z plane at *x/L_t_* = 0.16 (plane A2 marked in Figure 4 and Figure 10) for different Reynolds numbers. At *Re* = 0.01, M-4 shows the flow moving parallel to the wall, and there is no flow disturbance. However, at *Re* = 40, two symmetrical counter-rotating vortices are seen. These vortices get stronger with the increase in Reynolds number, as shown at *Re* = 120 with the additional vortices. Contrarily, velocity vectors in M-10 show a saddle-shaped flow structure even at the lowest Reynold number (*Re* = 0.01). This is the reason for the high mixing performance of M-10 in the low-*Re* range, which is discussed in Section 4.2.1. This result is in line with the observation in previous studies [84,87] that the saddle-shaped flow structure enhances mixing by stretching and folding of the fluid interfaces. 

**Figure 16:**
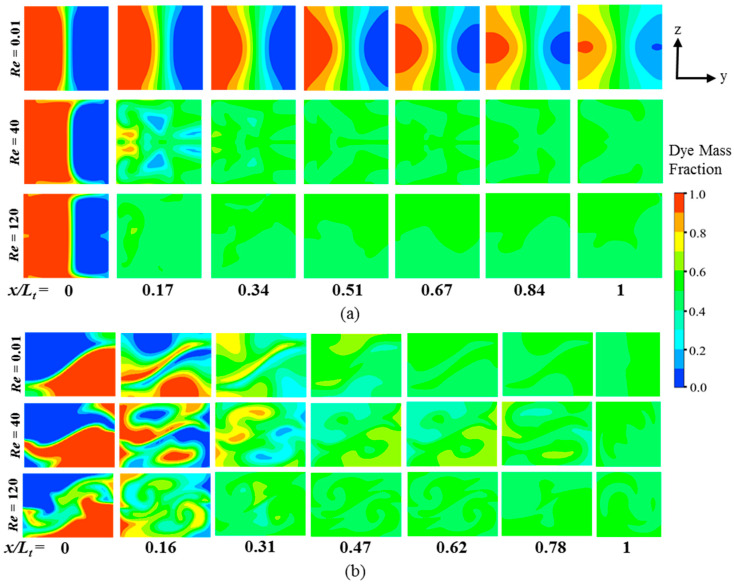
Dye concentration distributions at different Reynolds numbers: (**a**) M-4; and (**b**) M-10.

Figure 16 shows the dye concentrations on y-z planes of M-4 and M-10 for different Reynolds numbers. The progress of homogenization of the concentration in M-4 is much slower than that in M-10 at *Re* = 0.01. As shown in Figure 15, the fluids are mixed by pure diffusion in M-4, while M-10 shows a strong secondary flow structure even at this low Reynolds number, which is effective in promoting mixing. However, at high Reynolds numbers, the progress of mixing in M-4 is much faster than that in M-10 due to the occurrence of flow disturbances in the form of vortices (Figure 15) and high-inertia collisions of the streams on their recombinations as discussed in [77].

A selection procedure for micromixers is suggested as follows. Once the Reynolds number is fixed depending on the application, the micromixers showing preferred mixing index level should be selected considering ease of fabrication (2D or 3D). Pressure drop should also be considered as a vital factor in the case where the deformation of a sample will impact the outcome of the process, such as in biological applications. After narrowing down the micromixer types, mixing cost should be checked for the final selection.

## 5. Conclusions

Ten typical micromixers representing five different mixing mechanisms were analyzed quantitatively using Navier-Stokes equations under same working fluids, flow conditions, and axial channel length across a wide range of Reynolds number (*Re* = 0.01–120). In the results, M-9 and M-10 showed the best overall mixing indices among all the tested micromixers over the entire range of *Re*, while M-4 showed the best overall mixing indices among the 2D micromixers. However, M-3 showed the best overall MC values among the 2-D micromixers due to its incredibly low pressure drop regardless of Reynolds number. Compared to M-9 and M-10, M-4 showed far lower mixing indices in the low-*Re* range, but was represented among the similar or better mixing indices in the intermediate- and high-*Re* ranges. M-9 showed lower mixing performance than M-10 in the low-*Re* range, but showed higher mixing indices in the intermediate-*Re* range. M-10 showed the best overall MC values among the tested micromixers, which were slightly higher than those of M-9. The worst mixing performance was obtained by M-3 in the low-*Re* range, but by M-8 in the high-*Re* range. The stretching and folding of the fluid streams around the hyperbolic points created by the saddle-shaped flow structure in the crossing channel are more effective for mixing as compared to those generated by the grooves and barriers. Furthermore, the chaotic advection by crossing channels is generated over the entire range of Reynolds numbers, while for the other 2D and 3D channel designs, it is observed only at higher Reynolds numbers. In the crossing-channel micromixers, mixing does not depend upon inertial force, as indicated by good mixing even at low Reynolds numbers. Hence, these micromixers can also be applied to mix fluids having high viscosity. 

Therefore, among the tested micromixers, the Tesla structure micromixer (M-4) is recommended in the intermediate- and high-*Re* ranges considering high mixing performance and easy fabrication due to the planar structure, unless pressure drop is critical. The 3D micromixers, M-9 and M-10 are recommended in the low-*Re* range considering their excellent mixing performance. But, the fabrication of their two-layer structures with the traditional photolithography process is a challenging task due to the misalignment issue of the top and bottom layers. The results of this comparative analysis are intended to provide guidance for the selection of effective micromixers under different flow conditions in various microfluidic applications.

## Figures and Tables

**Table 1:** Types of micromixer designs.

Type	Micromixer Design	Mixing Mechanism	Selected Micromixer
1	2D designs using serpentine, spiral, curved helical channels [30,31,32,33,34,35,36,37,38,39,40,41]	Inertial force (secondary flow, Dean vortex)	M-1 [40], M-2 [41]
2	2D designs with SAR structures [68,69,70,71,72,73,74,75,76,77,78,79]	Inertial force, SAR	M-3 [68], M-4 [77]
3	3D design with serpentine and/or SAR structures [42,43,44,45,46,47,48,49,59,60,61,62,63,64,65,66,67,80,81]	Inertial force, chaotic mixing, multi-lamination	M-5 [45], M-6 [59], M-7 [81]
4	3D design with patterned grooves [29,50,51,52,53,54,55,56,57,58]	Inertial force, chaotic mixing	M-8 [58]
5	3D designs with SAR two-layer crossing channels [82,83,84,85,86,87,88,89]	Chaotic mixing, multi-lamination	M-9 [84], M-10 [87]

**Table 2:** Selected micromixers.

Micromixer Designation	Micromixer	Geometry (Type in Table 1)	Designers [Ref.]	Year
M-1	Curved micromixer	2D serpentine (1)	Hossain et al. [40]	2009
M-2	Curved micromixer with grooves	2D serpentine (1)	Alam and Kim [41]	2012
M-3	Modified P-SAR (planar SAR) micromixer with dislocation sub-channels	2D SAR (2)	Li et al. [68]	2013
M-4	Modified Tesla micromixer	2D SAR (2)	Hossain et al. [77]	2010
M-5	3D serpentine micromixer	3D serpentine (3)	Ansari and Kim [45]	2009
M-6	3D serpentine SAR micromixer	3D serpentine SAR (3)	Hossain and Kim [59]	2015
M-7	Improved serpentine laminating micromixer	3D serpentine SAR (3)	Park et al. [81]	2008
M-8	Barrier embedded chaotic micromixer	3D grooves (4)	Kim et al. [58]	2004
M-9	Chaotic micromixer with two-layer crossing microchannels	3D SAR (5)	Xia et al. [84]	2005
M-10	Chaotic micromixer with two-layer serpentine crossing microchannels	3D serpentine SAR (5)	Hossain et al. [87]	2017

**Table 3:** Optimum numbers of grid nodes selected through grid refinement tests at *Re* = 40.

Micromixer	Optimum Number of Meshes	Number of Finer Meshes	% Deviation of Mixing Index between the Optimum and Finest Meshes	Micromixer	Optimum Number of Meshes	Number of Finer Meshes	% Deviation of Mixing Index between the Optimum and Finest Meshes
M-1	1.81 × 10^6^	2.04 × 10^6^	1.39	M-6	2.07 × 10^6^	2.34 × 10^6^	0.10
M-2	2.07 × 10^6^	2.41 × 10^6^	1.16	M-7	2.19 × 10^6^	2.55 × 10^6^	0.54
M-3	1.55 × 10^6^	2.08 × 10^6^	0.94	M-8	2.21 × 10^6^	2.53 × 10^6^	1.05
M-4	2.16 × 10^6^	2.33 × 10^6^	0.20	M-9	1.90 × 10^6^	2.03 × 10^6^	0.10
M-5	1.79 × 10^6^	1.96 × 10^6^	0.10	M-10	1.61 × 10^6^	1.80 × 10^6^	0.11

**Table 4:** Mixing index at the exit.

Micromixer	Mixing Index at the Exit							
	Low-*Re* Range			Intermediate-*Re* Range		High-*Re* Range		
	*Re* = 0.01	*Re* = 0.1	*Re* = 1	*Re* = 20	*Re* = 40	*Re* = 60	*Re* = 80	*Re* = 120
M-1	0.560	0.250	0.224	0.572	0.793	0.979	0.990	0.998
M-2	0.554	0.244	0.221	0.694	0.858	0.974	0.997	0.997
M-3	0.268	0.141	0.125	0.241	0.422	0.657	0.828	0.890
M-4	0.465	0.255	0.203	0.883	0.999	0.999	0.999	0.999
M-5	0.546	0.361	0.377	0.999	0.999	0.999	0.999	0.999
M-6	0.649	0.506	0.472	0.999	0.999	0.999	0.999	0.999
M-7	0.904	0.594	0.537	0.567	0.733	0.856	0.909	0.963
M-8	0.310	0.238	0.226	0.250	0.284	0.310	0.337	0.401
M-9	0.939	0.909	0.905	0.934	0.972	0.987	0.998	0.996
M-10	0.970	0.926	0.915	0.901	0.929	0.995	0.973	0.996

**Table 5:** Pressure drop through micromixer.

Micromixer	Pressure Drop (Pa)							
	Low-*Re* Range			Intermediate-*Re* Range		High-*Re* Range		
	*Re* = 0.01	*Re* = 0.1	*Re* = 1	*Re* = 20	*Re* = 40	*Re* = 60	*Re* = 80	*Re* = 120
M-1	2.95 × 10^0^	2.95 × 10^1^	2.95 × 10^2^	6.77 × 10^3^	1.63 × 10^4^	2.81 × 10^4^	4.18 × 10^4^	7.70 × 10^4^
M-2	2.87 × 10^0^	2.87 × 10^1^	2.87 × 10^2^	6.64 × 10^3^	1.62 × 10^4^	2.80 × 10^4^	4.15 × 10^4^	7.68 × 10^4^
M-3	3.58 × 10^−1^	3.72 × 10^0^	3.73 × 10^1^	8.30 × 10^2^	1.95 × 10^3^	3.39 × 10^3^	5.14 × 10^3^	9.72 × 10^3^
M-4	1.03 × 10^0^	1.03 × 10^1^	1.04 × 10^2^	3.53 × 10^3^	1.20 × 10^4^	2.58 × 10^4^	4.50 × 10^4^	9.70 × 10^4^
M-5	7.94 × 10^−1^	7.94 × 10^0^	7.96 × 10^1^	2.44 × 10^3^	7.12 × 10^3^	1.39 × 10^4^	2.19 × 10^4^	4.32 × 10^4^
M-6	1.67 × 10^1^	1.67 × 10^2^	1.68 × 10^3^	5.32 × 10^4^	1.57 × 10^5^	3.10 × 10^5^	5.15 × 10^5^	1.09 × 10^6^
M-7	2.46 × 10^0^	2.46 × 10^1^	2.47 × 10^2^	5.27 × 10^3^	1.16 × 10^4^	1.95 × 10^4^	2.90 × 10^4^	5.49 × 10^4^
M-8	2.09 × 10^0^	2.12 × 10^1^	2.12 × 10^2^	4.29 × 10^3^	8.73 × 10^3^	1.33 × 10^4^	1.79 × 10^4^	2.75 × 10^4^
M-9	1.78 × 10^−1^	1.78 × 10^0^	1.78 × 10^1^	4.42 × 10^2^	1.16 × 10^3^	2.21 × 10^3^	3.59 × 10^3^	7.37 × 10^3^
M-10	1.63 × 10^−1^	1.63 × 10^0^	1.63 × 10^1^	3.90 × 10^2^	9.94 × 10^2^	1.84 × 10^3^	2.93 × 10^3^	5.90 × 10^3^

**Table 6:** Mixing cost.

Micromixer	Mixing Cost, *MC* (Pa^−1^)							
	Low-*Re* Range			Intermediate-*Re* Range		High-*Re* Range		
	*Re* = 0.01	*Re* = 0.1	*Re* = 1	*Re* = 20	*Re* = 40	*Re* = 60	*Re* = 80	*Re* = 120
M-1	1.90 × 10^−1^	8.47 × 10^−3^	7.58 × 10^−4^	8.45 × 10^−5^	4.85 × 10^−5^	3.48× 10^−5^	2.39 × 10^−5^	1.30 × 10^−5^
M-2	1.93 × 10^−1^	8.48 × 10^−3^	7.67 × 10^−4^	1.05 × 10^−4^	5.29 × 10^−5^	3.47× 10^−5^	2.40 × 10^−5^	1.30 × 10^−5^
M-3	7.51 × 10^−1^	3.79 × 10^−2^	3.36 × 10^−3^	2.90 × 10^−4^	2.16 × 10^−4^	1.94× 10^−4^	1.61 × 10^−4^	9.15 × 10^−5^
M-4	4.50 × 10^−1^	2.47 × 10^−2^	1.96 × 10^−3^	2.50 × 10^−4^	8.36 × 10^−5^	3.87× 10^−5^	2.22 × 10^−5^	1.03 × 10^−5^
M-5	6.88 × 10^−1^	4.55 × 10^−2^	4.74 × 10^−3^	4.09 × 10^−4^	1.40 × 10^−4^	7.18× 10^−5^	4.57 × 10^−5^	2.32 × 10^−5^
M-6	3.89 × 10^−2^	3.03 × 10^−3^	2.82 × 10^−4^	1.88 × 10^−5^	6.38 × 10^−6^	3.22× 10^−6^	1.94 × 10^−6^	9.20 × 10^−7^
M-7	3.67 × 10^−1^	2.41 × 10^−2^	2.18 × 10^−3^	1.07 × 10^−4^	6.29 × 10^−5^	4.40× 10^−5^	3.13 × 10^−5^	1.76 × 10^−5^
M-8	1.48 × 10^−1^	1.12 × 10^−2^	1.07 × 10^−3^	5.81 × 10^−5^	3.25 × 10^−5^	2.34× 10^−5^	1.88 × 10^−5^	1.46 × 10^−5^
M-9	5.28 × 10^0^	5.11 × 10^−1^	5.08 × 10^−2^	2.12 × 10^−3^	8.38 × 10^−4^	4.47× 10^−4^	2.78 × 10^−4^	1.35 × 10^−4^
M-10	5.95 × 10^0^	5.68 × 10^−1^	5.60 × 10^−2^	2.31 × 10^−3^	9.34 × 10^−4^	5.41× 10^−4^	3.32 × 10^−4^	1.69 × 10^−4^

## Nomenclature

*c*	mass fraction
*D*	diffusion coefficient (m^2^/s)
*L_i_*	length of initial part of main channel (µm)
*L_e_*	exit channel length (µm)
*L_t_*	total length of the micromixer (µm)
*M*	mixing index
*M_0_*	mixing index at the exit
*N*	number of sampling points
*P*	pressure drop (Pa)
*Pi*	pitch length (µm)
*Re*	Reynolds number
*SAR* *U*	split and recombine average inlet velocity (m/s)
*W*	width of main channel (µm)
x, y, z	Cartesian coordinates
**Greek Symbols**	
*µ*	fluid dynamic viscosity (kg·m^−1^·s^−1^)
*ν*	fluid Kinematic viscosity (m^2^/s)
*ρ*	fluid density (kg/m^3^)
*σ*	standard deviation
**Subscripts**	
*i*	sampling point or fluid component
*m*	optimal mixing
*max*	maximum value
*x*	axial distance
